# Transcriptomic and targeted metabolomic analyses provide insights into the flavonoids biosynthesis in the flowers of *Lonicera macranthoides*

**DOI:** 10.1186/s12896-024-00846-5

**Published:** 2024-04-12

**Authors:** Ling Ling Lv, Li Yun Li, Long Qian Xiao, Jian Hui Pi

**Affiliations:** Key Laboratory of Research and Utilization of Ethnomedicinal Plant Resources of Hunan Province, Key Laboratory of Hunan Higher Education for Western Hunan Medicinal Plant and Ethnobotany, Huaihua University, 418008 Huaihua, China

**Keywords:** *Lonicera macranthoides*, Transcriptome, Targeted metabolome, Flavonoid biosynthesis, Flavone, Flavonol, Anthocyanin, Flower

## Abstract

**Background:**

Flavonoids are one of the bioactive ingredients of *Lonicera macranthoides* (*L. macranthoides*), however, their biosynthesis in the flower is still unclear. In this study, combined transcriptomic and targeted metabolomic analyses were performed to clarify the flavonoids biosynthesis during flowering of *L. macranthoides*.

**Results:**

In the three sample groups, GB_vs_WB, GB_vs_WF and GB_vs_GF, there were 25, 22 and 18 differentially expressed genes (DEGs) in flavonoids biosynthetic pathway respectively. A total of 339 flavonoids were detected and quantified at four developmental stages of flower in *L. macranthoides*. In the three sample groups, 113, 155 and 163 differentially accumulated flavonoids (DAFs) were detected respectively. Among the DAFs, most apigenin derivatives in flavones and most kaempferol derivatives in flavonols were up-regulated. Correlation analysis between DEGs and DAFs showed that the down-regulated expressions of the *CHS, DFR, C4H, F3’H*, *CCoAOMT_*32 and the up-regulated expressions of the two *HCT*s resulted in down-regulated levels of dihydroquercetin, epigallocatechin and up-regulated level of kaempferol-3-O-(6’’-O-acetyl)-glucoside, cosmosiin and apigenin-4’-O-glucoside. The down-regulated expressions of *F3H* and *FLS* decreased the contents of 7 metabolites, including naringenin chalcone, proanthocyanidin B2, B3, B4, C1, limocitrin-3,7-di-O-glucoside and limocitrin-3-O-sophoroside.

**Conclusion:**

The findings are helpful for genetic improvement of varieties in *L.macranthoides*.

**Supplementary Information:**

The online version contains supplementary material available at 10.1186/s12896-024-00846-5.

## Background

*Lonicera macranthoides* Hand. Mazz (*L. macranthoides*), the most widely cultivated species of Lonicerae flos grown in south China, is one of the traditional Chinese medicinal plants. Lonicerae flos was separated from honeysuckle (*Lonicera japonica*) grown in north China in the Chinese Pharmacopoeia in 2005. The main medicinal ingredients of honeysuckle are luteoloside and chlorogenic acid, and the market price of its fresh flowers is about 360 yuan per kilogram [1]. The main medicinal ingredients of *L. macranthoides* are phenolic acids, flavonoids and carotenoids, especially chlorogenic acid, and the market price of its fresh flower buds or early flowers is about 130 yuan per kilogram [1]. Low prices due to differences in medicinal ingredients hinder the sustainable development of the *L. macranthoides* industry.

Flavonoids, including flavonols, flavones, flavanols, isoflavones, flavanones and anthocyanins, are a major class of secondary metabolites existing in all higher plants [2]. They are not only very important pigments that contribute to a range of flower colors from yellow to purple [3], but also important bioactive ingredients. They have antibacterial, anti-inflammatory, antioxidant, anticancer; antiviral and antimicrobial and other functions [4–7], and can be used for the treatment of diseases such as throat arthralgia, erysipelas, heat toxic blood dysentery, wind heat cold, warm heat disease, etc. [8–9]. They also played important roles in the treatment of the severe acute respiratory syndrome (2003) and COVID-19 (2019) outbroke in China. Twenty-one flavonoids have been isolated from *L. macranthoides*, including kaempferol-3-O-β-D-glucoside, quercetin-3-O-β-D-glucoside and luteolin-7-O-β-D-glucoside, etc [10–13]. The biosynthetic pathways of flavonoids in apple [14], *Salvia miltiorrhiza* [15], *Arabidopsis thaliana* and maize [16] have been well studied.

Compared with honeysuckle, the research of *L.macranthoides* in all aspects lags behind seriously. At present, the researches on *L.macranthoides* mainly focuse on the determination of medicinal ingredients, RNA sequencing, cloning and verification of functional genes [17–19]. These studies are fragmentary and lack systematicness. In recent years, the regulation mechanism of functional ingredients have been elucidated through the combined analysis of transcriptome and metabolome in honeysuckle [20], cucumber [21], dendrobium [22] and Longan [23]. Pan et al. [24] reported the regulatory mechanism of chlorogenic acid (CGA) biosynthesis in *L.macranthoides* and the effect of corolla dehiscence on the quality of medicinal function. In this work, combined transcriptomic and metabolomic analyses were performed to clarify the flavonoids biosynthesis in the flowers of *L.macranthoides*. The findings are helpful for breeding new varieties of *L.macranthoides* with high content of medicinal ingredients and increasing the economic income of growers.

## Results

### Transcriptome analysis

To evaluate the gene expression profile in *L. macranthoides* flowers at GB, WB, WF and GF, 12 cDNA libraries were constructed and sequenced. The raw data has been deposited in the China National GeneBank DataBase (CNGBdb, https://db.cngb.org/) with accession code CNP0003782. A total of 158.96 Gb clean data was obtained and the GC content of each sample was more than 44.3%. The percentages of Phred-like quality score at the Q30 level (an error probability of 1%) ranged from 90.94 to 93.72%.

**Fig. 1:**
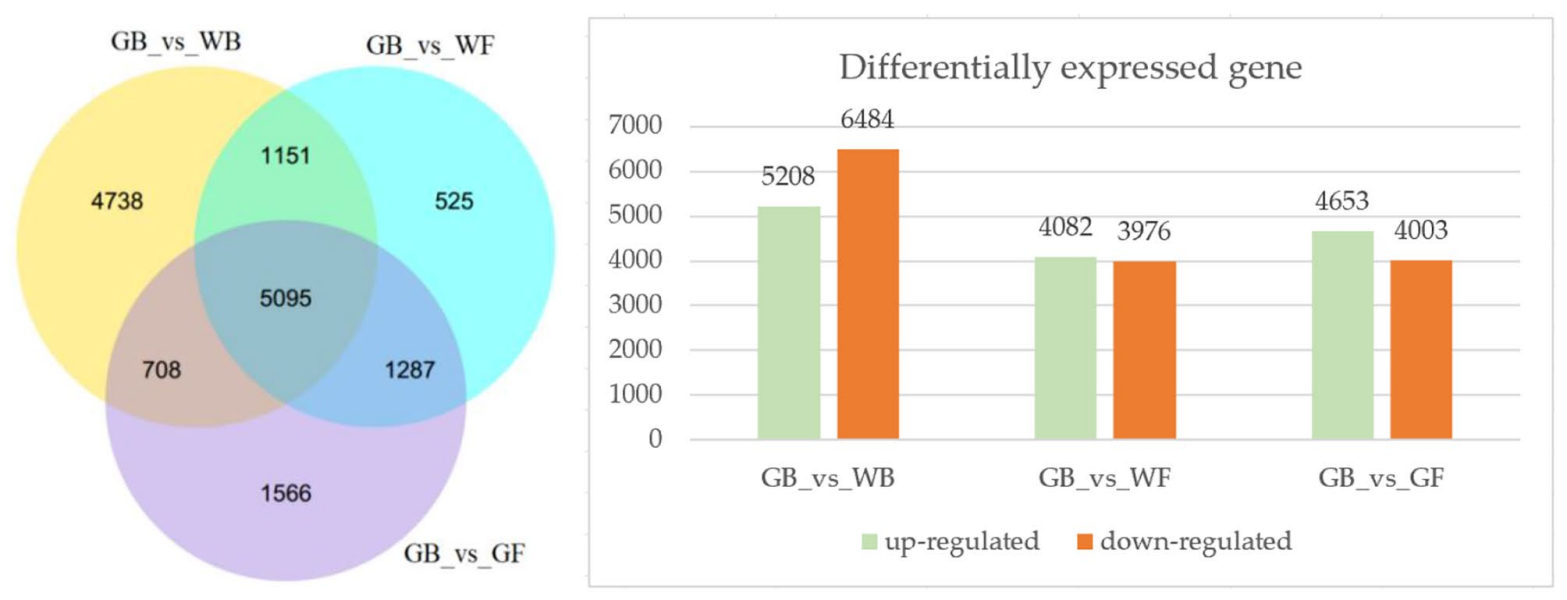
Venn Diagram and the up- and down-regulated differentially expressed genes in the three sample groups. GB, green flower bud; WB, white flower bud; WF, white flower; GF, golden flower

There were 11,692, 8,058, and 8,656 differentially expressed genes (DEGs) in the three sample groups (GB_vs_WB, GB_vs_WF and GB_vs_GF) respectively (Fig. 1). Among the three sample groups, the most DEGs were found in GB_vs_WB, with 5,208 DEGs up-regulated and 6,484 DEGs down-regulated. Venn diagram analysis showed that 5,095 DEGs were common to all three sample groups (Fig. 1).

Gene ontology (GO) classification analysis assigned 57,129, 29,156 and 36,957 unigenes to the three major categories (Biological Process, Cell Component and Molecular Functional), respectively (Supplementary Fig. S1). In the ‘Biological Process’ category, the subcategory with the most unigenes was ‘metabolic process’ (GO:0008152, 14,175 unigenes), followed by ‘cellular process’ (GO:0009987, 13,316 unigenes) and ‘biological regulation’ (GO:0065007, 3,948 unigenes). In the ‘Molecular Function’ category, the most enriched subcategory was ‘binding’ (GO:0005488, 18,510 unigenes). In addition, ‘nucleic acid binding transcription factor activity’ (GO:0001071) included 678 unigenes and ‘molecular function regulator’ (GO:0098772) included 485 unigenes.

**Fig. 2:**
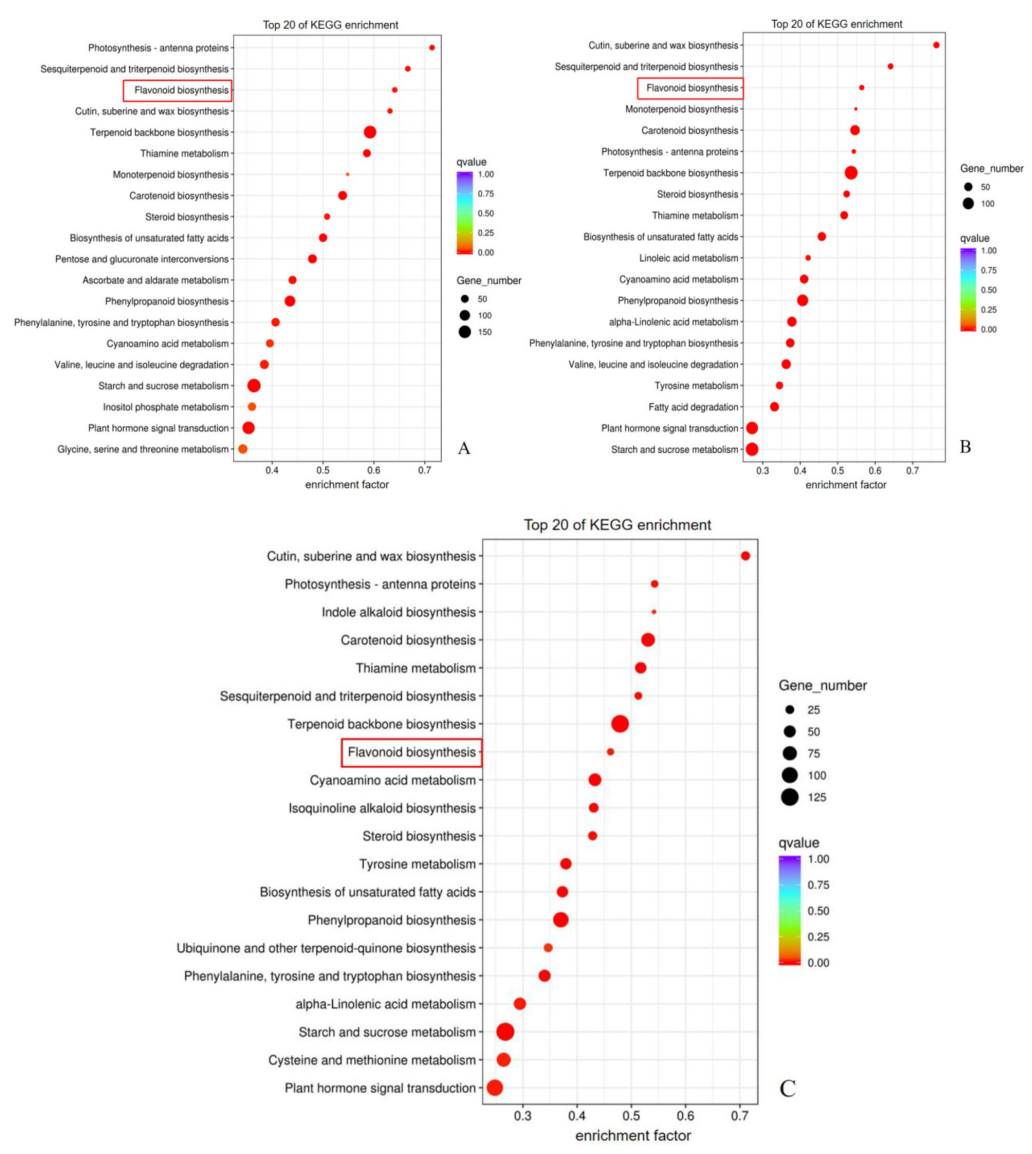
KEGG pathway classification of differentially expressed genes in the three sample groups of *L. macranthoides.***(A)** In GB_vs_WB. **(B)** In GB_vs_WF. **(C)** In GB_vs_GF.

All DEGs were BLAST against the KEGG Ortholog database [25] and the top 20 KEGG categories in the three sample groups were presented respectively in Fig. 2A-C. In GB_vs_WB, GB_vs_WF and GB_vs_GF, there were 25, 22 and 18 DEGs in flavonoids biosynthesis (ko00941) respectively. Among the DEGs in flavonoids biosynthetic pathway, *PAL, C4H*, two *CHS*s, *CHI, F3H, F3’H*, two *DFR*s, *CCoAOMT_*32, *ANS* and *ANR* were all down-regulated, while *FLS_*73, *C4H_*90, *CCoAOMT_*73 and three *HCT*s were up-regulated in the three sample groups.

### Validation of RNA-seq data by qPCR analysis

**Fig. 3:**
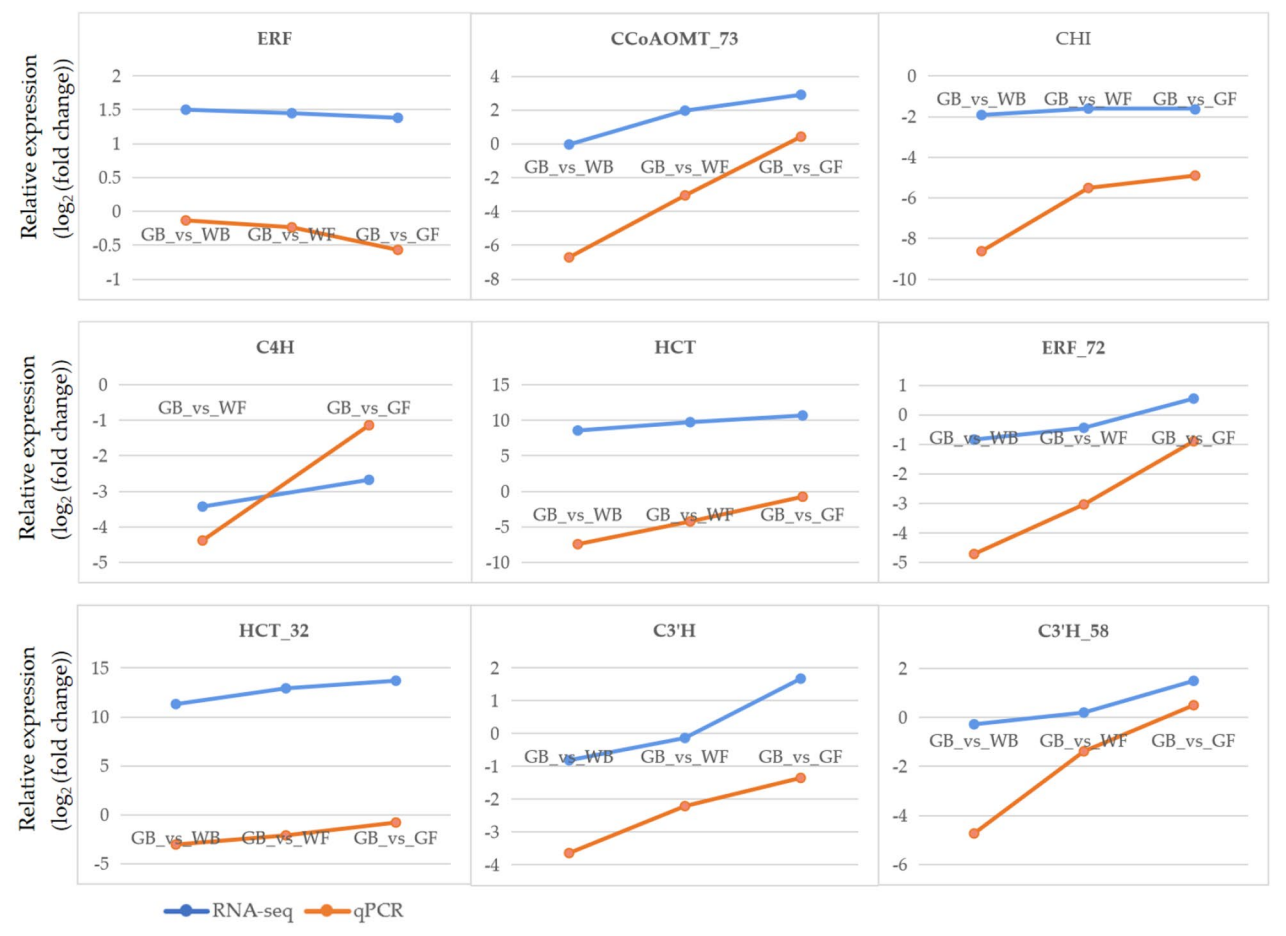
qPCR validation of RNA-seq data using 9 related genes in *L. macranthoides. 18 S rRNA* expression values were used as the internal reference. Values are the means ± SD of three biological replicates. GB, green flower bud; WB, white flower bud; WF, white flower; GF, golden flower

To validate the RNA-Seq data, 9 DEGs (two transcription factors and 7 genes in flavonoids biosynthetic pathway) were selected and their expression levels at GB, WB, WF and GF were analyzed by qPCR. As shown in Fig. 3, the expressions of 8 DEGs were higher in RNA-seq than in qPCR. Though the expression levels of these DEGs were different, the trends in gene expression of qPCR were highly consistent with those of RNA-seq, which suggested that RNA-seq data was credible and could be used for subsequent experiments.

### Flavonoids assay

**Fig. 4:**
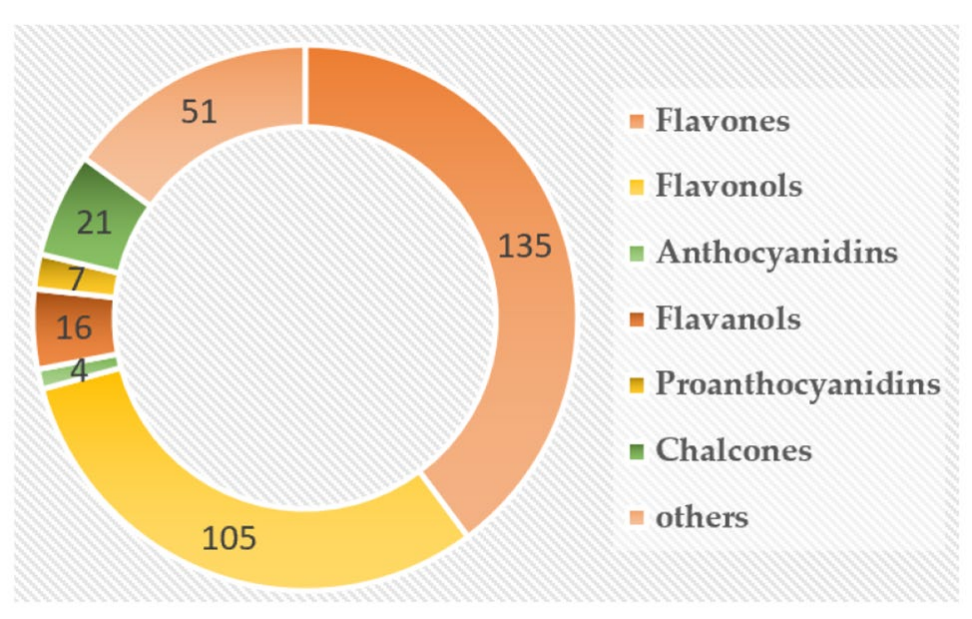
The total flavonoids detected in the samples

**Fig. 5:**
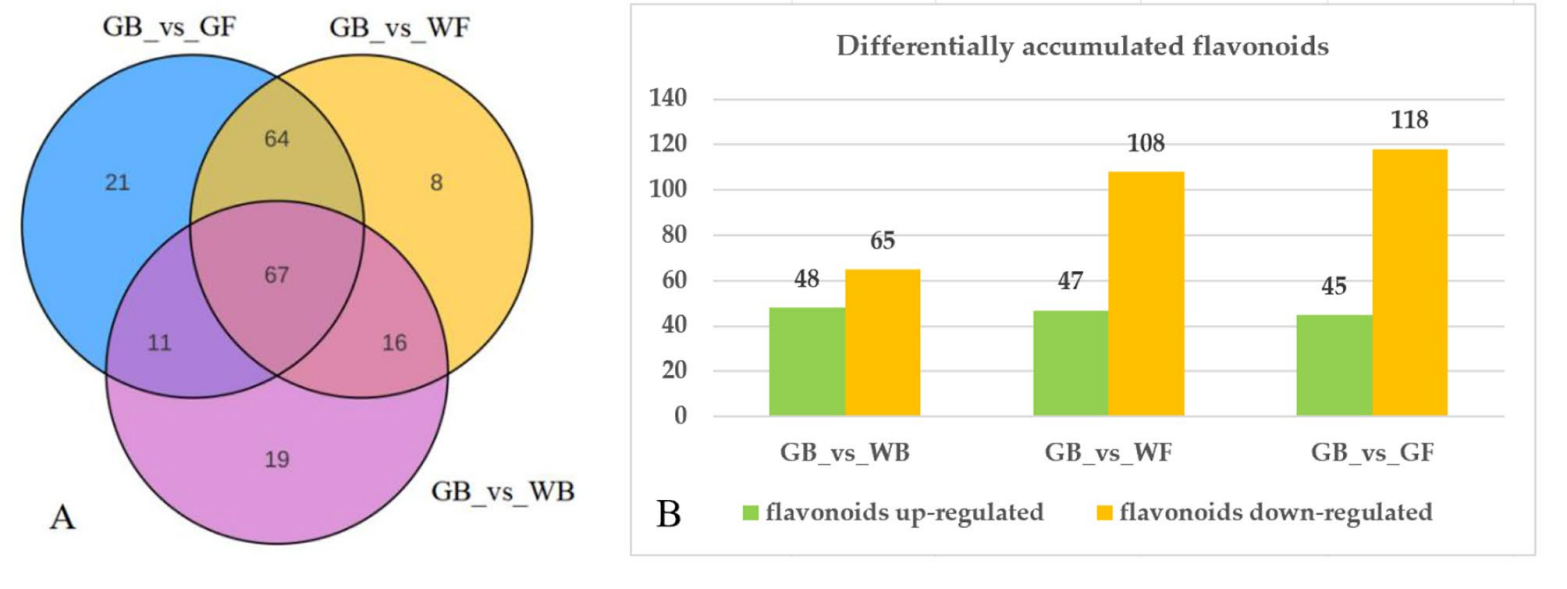
The differentially accumulated flavonoids in the three sample groups. **(A)** Venn diagram depicting the shared and specific flavonoids. **(B)** The up- and down-regulated flavonoids. GB, green flower bud; WB, white flower bud; WF, white flower; GF, golden flower

**Fig. 6:**
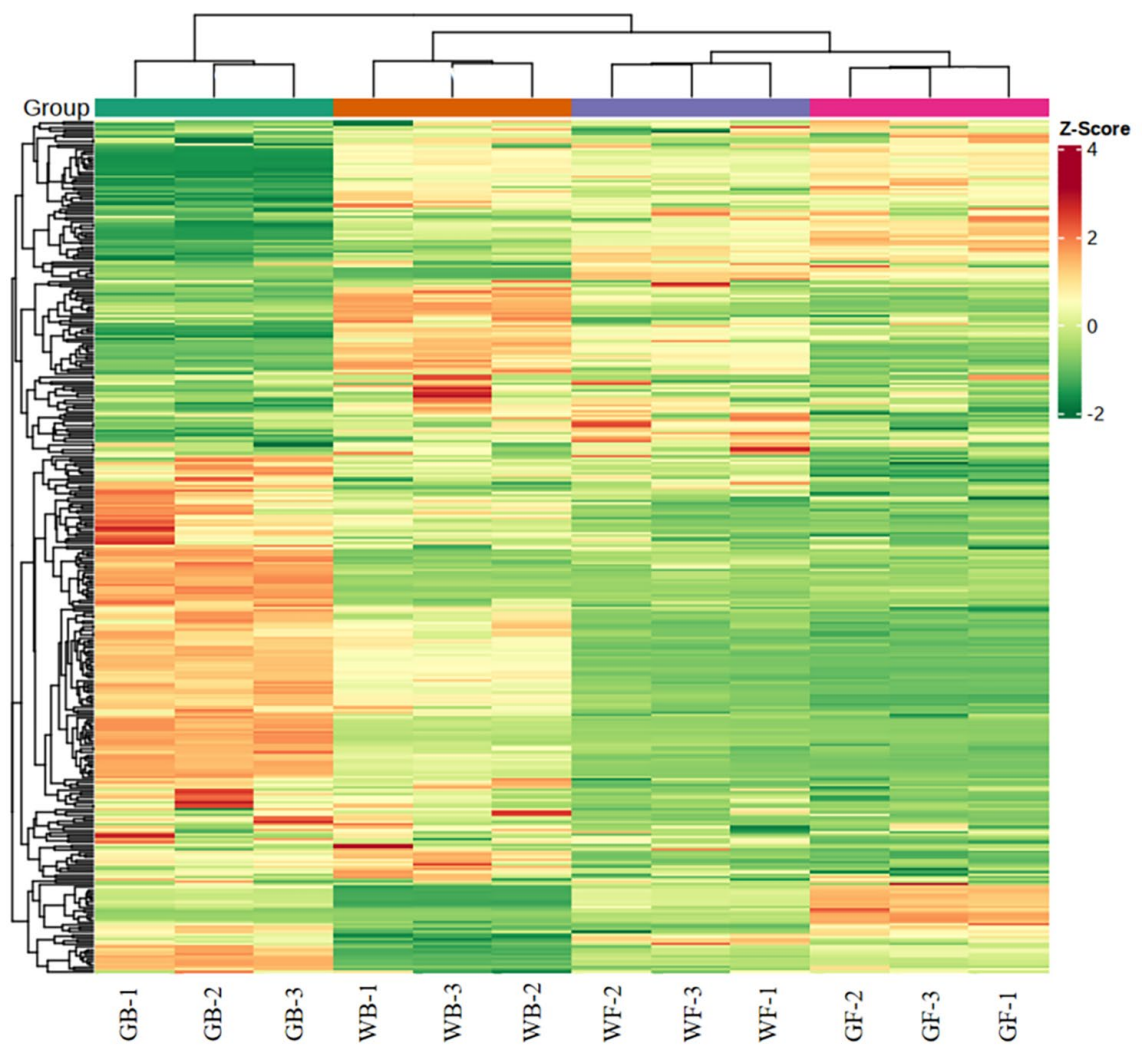
Clustering heat map of the flavonoids detected in the total samples. Each sample is visualized in a single column, and each metabolite is represented in a single row. Red indicates high abundance of metabolites, while green indicates low abundance. GB, green flower bud; WB, white flower bud; WF, white flower; GF, golden flower

A total of 339 flavonoids were detected and quantified at four developmental stages of flower in *L. macranthoides*, including 135 flavones, 105 flavonols, 16 flavanols, 4 anthocyanins, 7 proanthocyanidins, 21 chalcones and 51 other flavonoids (Fig. 4). Among the three sample groups, the most differentially accumulated flavonoids (DAFs) were found in GB_vs_GF, with 45 DAFs up-regulated and 118 DAFs down-regulated (Fig. 5A-B). Venn diagram analysis showed that 67 DAFs were common to all three sample groups (Fig. 5A). Based on the accumulation patterns of flavonoids, the clustering heat map was shown in Fig. 6. The biological replicates were all grouped together, indicating a high reliability of the metabolome data.

**Fig. 7:**
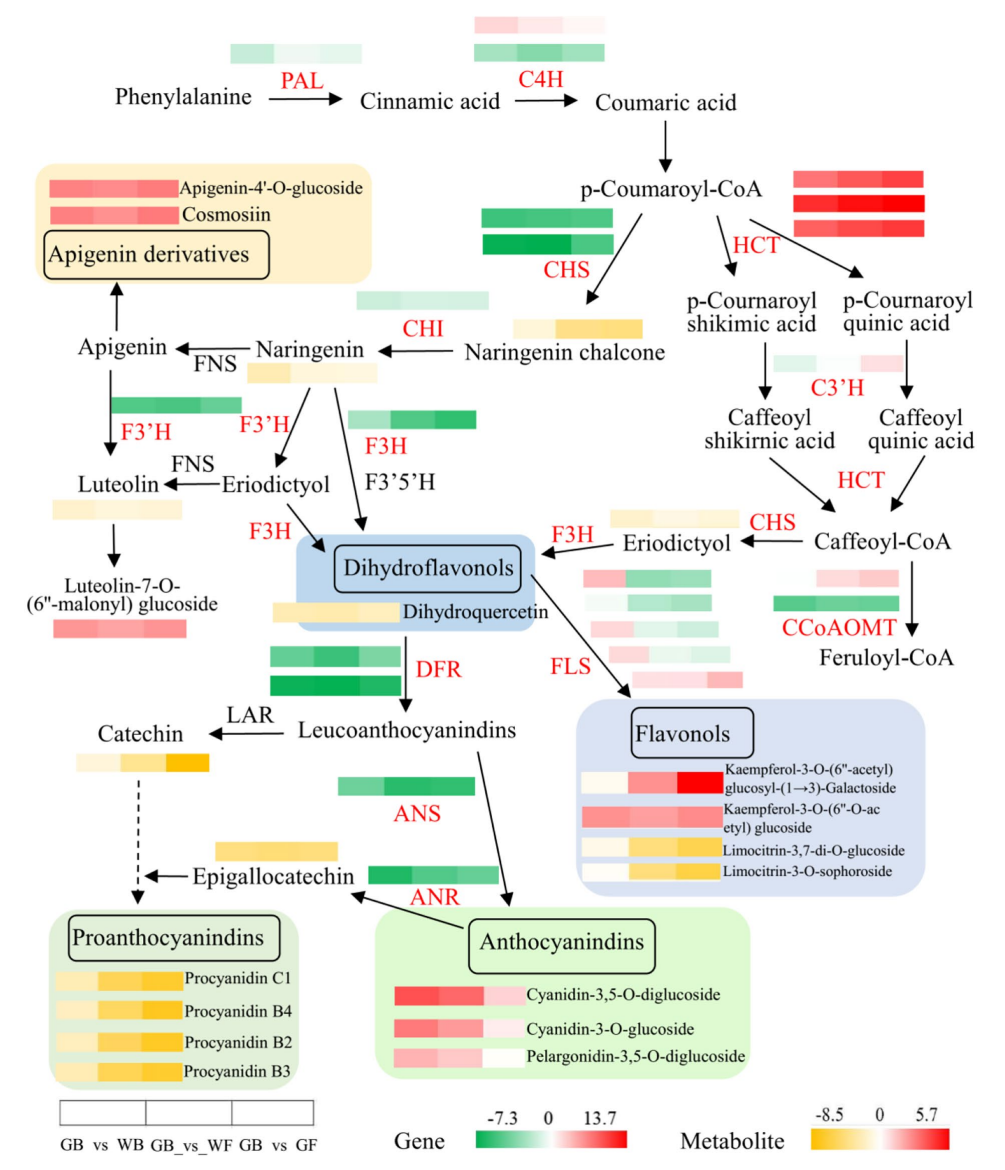
The pathway associated with flavonoid biosynthesis in *L. macranthoides* and heat maps of differentially expressed genes (DEGs) and differentially accumulated flavonoids in three sample groups. The red font represents DEG. ANS, anthocyanidin synthase; ANR, anthocyanidin reductase; CCoAOMT, caffeoyl-CoA O-methyl-transferase; CHI, chalcone isomerase; CHS, chalcone synthase; C4H, cinnamate-4-hydroxylase; C3’H, p-coumarate 3’-hydroxylases; DFR, dihydroflavonol reductase; F3’5’H, flavonoid 3’,5’- hydroxylase; F3’H, flavonoid 3’-hydroxylase; F3H, flavanone 3-hydroxylase; FLS, flavonol synthase; FNS, flavone synthase; HCT, hydroxycinnamoyl CoA shikimatelquinate hydroxycinnamoyltransferase; LAR, leucocyanidin reductase; PAL, phenylalanine ammonialyase; 4CL, 4-coumaric acid coenzyme a ligase

5,7,3’,4’-tetrahydroxy flavone (Luteolin) and its derivative of luteolin-7-O-(6’’-malonyl)-glucoside (LOG) were detected in the three sample groups. The former was all down-regulated, while the latter was all up-regulated (Fig. 7). Four and five other luteolin derivatives were detected in GB_vs_WF and GB_vs_GF respectively, all of them were down-regulated. Cyanidin-3,5-O-diglucoside and pelargonidin-3,5-O-diglucoside were detected in GB_vs_WB and GB_vs_WF, and they were all up-regulated. Cyanidin-3-O-glucoside was only detected in GB_vs_WB and also up-regulated (Fig. 7). In the three sample groups, a total of 61 differentially accumulated flavonols were detected. The up-regulated flavonols were mainly kaempferol derivatives, while the down-regulated flavonols were mainly limonin derivatives. Four differentially accumulated dihydroflavonols were detected, among which dihydroquercetin was down-regulated in all three sample groups. Seven differentially accumulated procyanidins, A1, B1, B2, B3, B4, C1, C2, were detected and procyanidin A1 was only found in GB_vs_GF. The other 6 procyanidins were detected in the three sample groups and their contents were all down-regulated. In addition, the DAFs of naringenin chalcone, naringenin, eriodictyol, catechin and epigallocatechin were all down-regulated in the three sample groups (Fig. 7).

### Correlation analysis between transcripts and flavonoids

**Fig. 8:**
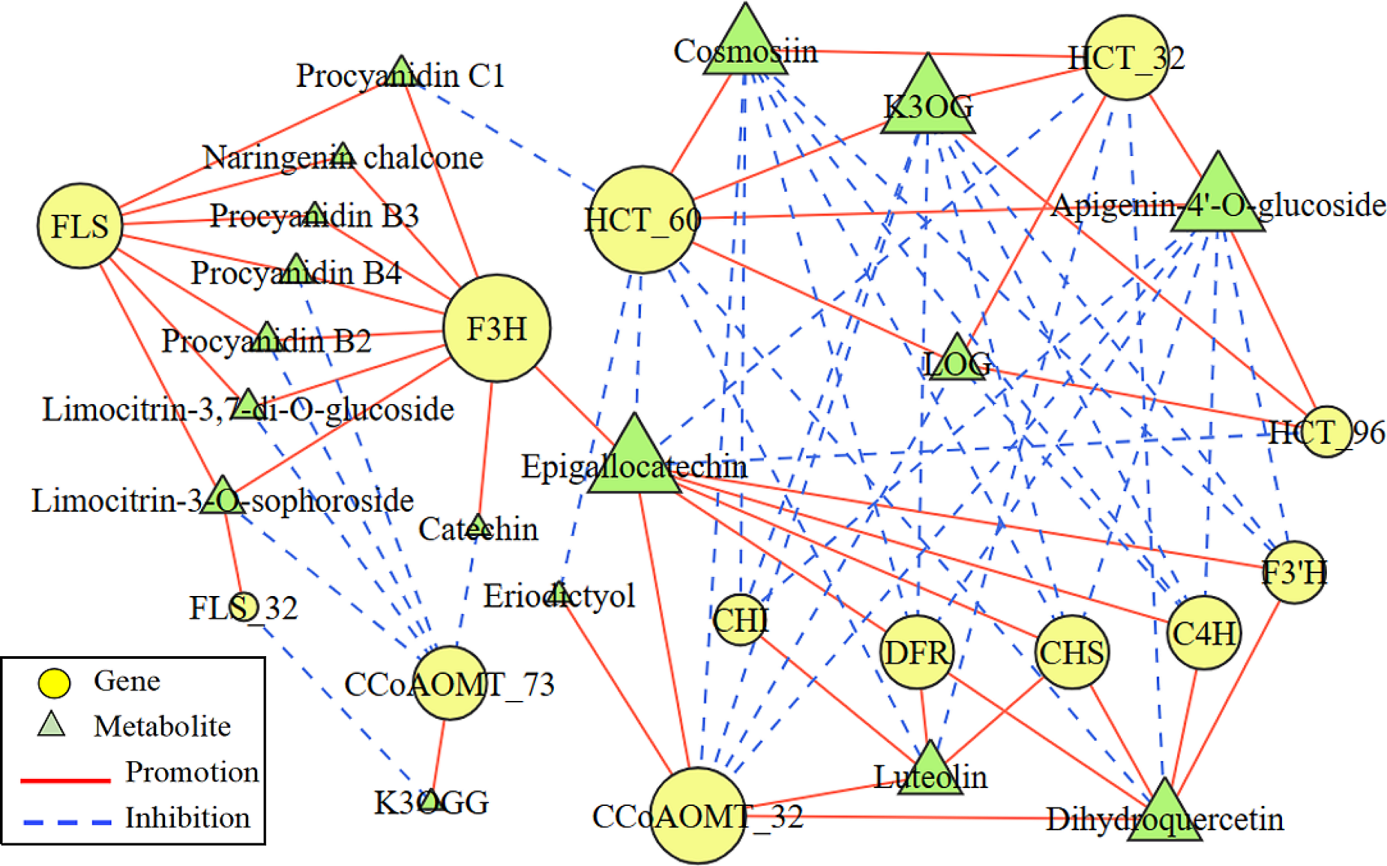
Connection network between genes and flavonoids. K3OG, Kaempferol-3-O-(6’’-O-acetyl)- glucoside; LOG, luteolin-7-O-(6’’-malonyl)-glucoside; K3OGG, Kaempferol-3-O-(6’’-acetyl)glucosyl- (1→3)-galactoside. The size of the triangle and the circle represents the number of genes or metabolites associated with them, respectively

Thirteen DEGs and 17 DAFs in the flavonoids biosynthetic pathways (Fig. 7) were obtained to organize an interaction network (Fig. 8) using Cytoscape 2.0 software. *CHS, DFR, C4H, F3’H* and *CCoAOMT_*32 were positively correlated with dihydroquercetin and epigallocatechin and negatively correlated with kaempferol-3-O-(6’’-O-acetyl)-glucoside (K3OG), apigenin-7-O-glucoside (Cosmosiin) and apigenin-4’-O-glucoside. The regulatory effects of *HCT*_60, *HCT*_32 and the above five genes were just opposite (Fig. 8). The down-regulated expressions of *F3H* and *FLS* decreased the contents of 7 metabolites, including naringenin chalcone, proanthocyanidin B2, B3, B4, C1, limocitrin-3,7-di-O- glucoside and limocitrin-3-O-sophoroside (Figs. 7 and 8). In addition, *F3H* was positively correlated with epigallocatechin and catechin. *HCT*_60 and *HCT*_32 were negatively correlated with luteolin, while *CHS, DFR, CCoAOMT*_32 and *CHI* were positively correlated with it. The flavonoids associated with *CCoAOMT*_32 and *CCoAOMT*_73, respectively, were completely different. There were similarities and differences among the flavonoids associated with the three *HCT*s respectively (Fig. 8).

## Discussion

Flavone is a major class of flavonoids and 135 flavones were detected in this work, 89 of which were DAFs. The contents of LOG, cosmosiin and apigenin-4’-O-glucoside increased in the three sample groups (Fig. 7), and their contents at GF were 5.29, 7.81 and 7.72 times of those at GB respectively. *HCT*_60 and *HCT*_32 were positively correlated with the accumulations of LOG, apigenin-4’-O-glucoside and cosmosiin, while *C4H* and *CCoAOMT*_32 were negatively regulated them (Fig. 8). In addition, apigenin-7-O-(2’’-glucosyl)arabinoside, apigenin-6-C-(2’’-xylosyl)-glucoside, apigenin-8-C-(2’’-xylosyl)-glucoside and apigenin-7-O-(6’’-acetyl)-glucoside were also up-regulated in the three sample groups. Tricin-7-O-(2’’-malonyl)rhamnoside (TMR) had the largest fold change of 56.13 in differentially accumulated flavone in GB_vs_GF. These suggested that the flavones of LOG, apigenin derivatives and TMR were one of the main medicinal ingredients in the late flowers of *L. macranthoides*. Yang et al. [26] reported that flavones had antibacterial, antioxidant and anticancer effects. Apigenin had been demonstrated to have medicinal functions in the treatment of cancer [27], epilepsy [28] and hyperlipemia [29].

Flavonols have the functions of antiallergy, anti-inflammation, anticancer and preventing diabetes [30–31]. FLS is a key enzyme in the synthesis of flavonols and different *FLS* genes may play the same or different roles [32]. In this study, *FLS* positively correlated with proanthocyanidins C1, B2, B3, B4, naringin chalcone and limonin derivatives, while *FLS*_32 negatively regulated kaempferol-3-O-(6’’-acetyl)glucosyl-(1→3)-galactoside (K3OGG). The other three *FLS*s showed no correlation with flavonoids accumulation (Fig. 8). Among the six *FLS*s in *Arabidopsis Thaliana*, *AtFLS1* plays an important role in flavonoid synthesis [33]. The up-regulated flavonols were mainly kaempferol derivatives, such as K3OGG, K3OG, kaempferol-3-O-galactoside-4’-O-glucoside and kaempferol-3-O-(6’’-malonyl)galactoside, which suggested that *FLS*s catalyzed dihydrokaempferol to form kaempferol, rather than dihydroquercetin to quercetin or dihydromyricetin to myricetin. The substrates of FLS are dihydroquercetin, dihydrokaempferol and dihydromyricetin, and the binding of FLS to the substrates is specific [34]. The content of K3OGG decreased slightly in GB_vs_WB, then increased sharply, and had the largest fold change of 52.60 in differentially accumulated flavonols in GB_vs_GF.

Anthocyanins have anti-inflammatory and antioxidant effects [4–5], and they are synthesized in the flavonoid pathway (Fig. 7). In this work, two differentially accumulated cyanidin derivatives, cyanidin-3,5-O-diglucoside and cyanidin 3-O-glucoside, and differentially accumulated pelargonidin-3,5-O-diglucoside were detected in GB_vs_WB and GB_vs_WF. Moreover, the contents of cyanidin derivatives were considerably higher than that of pelargonidin derivative. Yi et al. [23] reported the similar result in longan pericarp. The contents of the three anthocyanins reached the highest at WB and then decreased gradually (Fig. 7). Xue et al. [20] reported that anthocyanins were detected at S1 and S2 of early flowering and were not present at S3-S6. *DFR* has selective specificity for dihydrokaempferol, dihydroquercetin and dihydromyricetin [35]. In this work, it seems that *DFR*s atalyzed dihydroquercetin to produce cyanidin, rather than dihydromyricetin to delphinidin. Similar result was reported in *Ficus carica* L [36].. The expression of *CHS* was significantly correlated with anthocyanin accumulation [37]. Surprisingly, in this study, the expressions of key genes in anthocyanin biosynthetic pathway, such as *CHS, DFR, ANS*, etc. were all down-regulated, while the contents of the above three anthocyanins were up-regulated in GB_vs_WB and GB_vs_WF. Is there another anthocyanin biosynthetic pathway in the flowers of *L. macranthoides*?

HCT belongs to plant acyltransferase family and can catalyze a variety of substrates, including shikimic acid, 4-hydroxyphenyllactic acid, quinic acid, 4-hydroxyphenylethylamine and gentianic acid, to form ester or amide compounds [38]. *HCT* plays an important role in the biosynthesis of lignin and CGA [39–40]. In this work, *p*-coumaroyl-CoA was catalyzed by HCT, C3’H and CHS to synthesize eriodictyol, thus participating in flavonoid biosynthesis (Fig. 7). Although the expressions of three *HCT*s were significantly up-regulated, the content of eriodictyol was down-regulated (Fig. 7). It might be because most of the synthesized caffeoyl-CoA rapidly entered the biosynthesis of downstream metabolites, resulting in a decrease in the amount of eriodictyol.

In this work, transcriptomic and targeted metabolomic analyses provided insights into the flavonoids biosynthesis in the flowers of *L.macranthoides*. However, the specific regulatory mechanisms of flavonoids, including the interesting finding in the study, need a further research.

In short, the synthesis of flavonoids in the flowers of *L. macranthoides* is a dynamic balance of various regulatory networks. The same metabolic process may be regulated by multiple genes and a gene may be involved in the regulation of multiple metabolic processes. The ultimate medicinal value of *L. macranthoides* is determined by the whole gene regulation network, and is ultimately reflected by the types and contents of metabolites.

## Conclusions

Flavonoids are one of the bioactive ingredients in the flowers of *L. macranthoides*. In this study, correlation analysis between DEGs and DAFs showed that the down-regulated expressions of the *CHS, DFR, C4H, F3’H*, *CCoAOMT_*32 and the up-regulated expressions of the two *HCT*s resulted in up-regulated level of K3OG, cosmosiin and apigenin-4’-O-glucoside. The down-regulated expressions of *F3H* and *FLS* decreased the contents of 7 metabolites, including naringenin chalcone, proanthocyanidin B2, B3, B4, C1, limocitrin-3,7-di-O-glucoside and limocitrin-3-O-sophoroside. The results showed that the early flower buds mainly contained limonin derivatives, procyanidins, catechin, luteolin and cyanidin derivatives, while the late flowers mainly contained kaempferol derivatives, apigenin derivatives and TMR. These indicated that flavonoids exerting medicinal functions were different in flowers at different developmental stages. The findings are helpful for genetic improvement of varieties in *L.macranthoides*.

## Materials and methods

### Plant materials

**Fig. 9:**
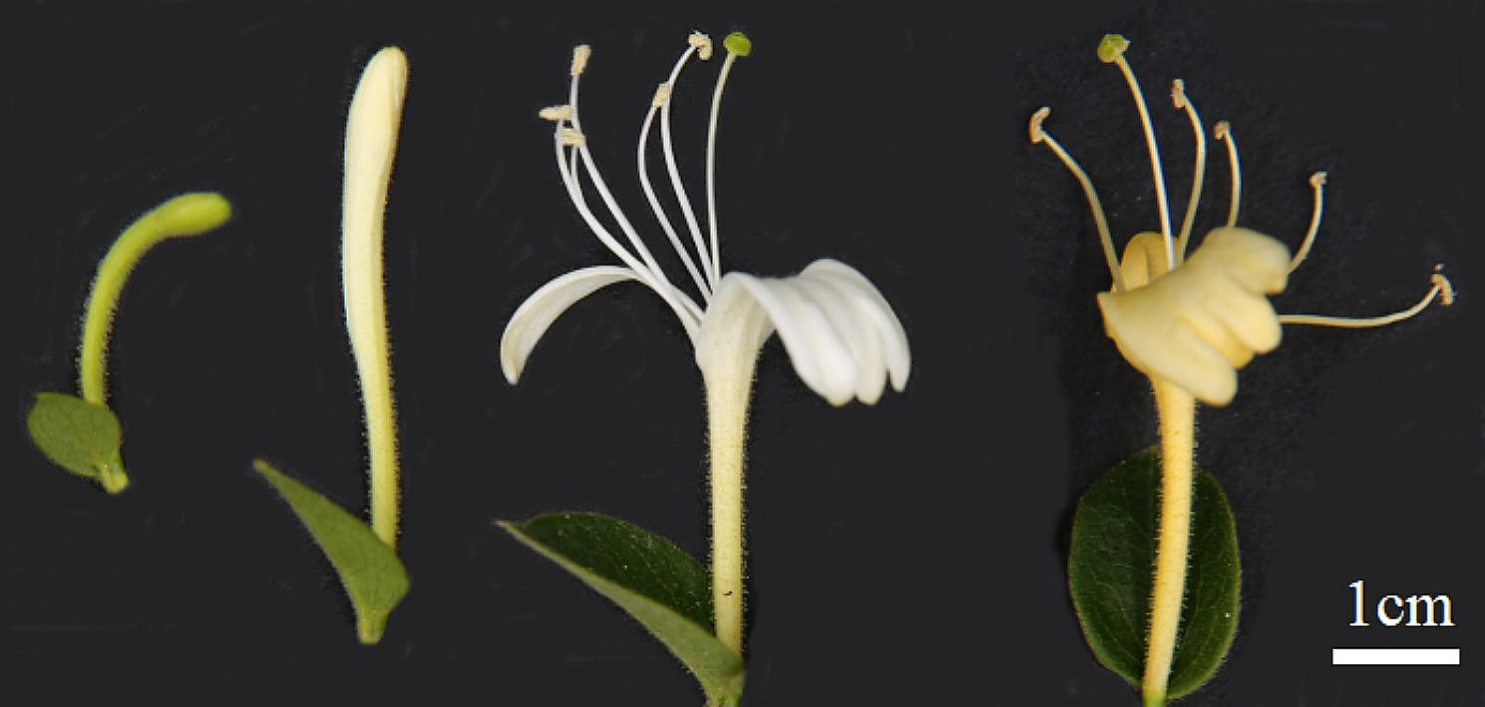
The four developmental stages of flower in *L. macranthoides.* From left to right, green flower bud (GB), white flower bud (WB), white flower (WF) and golden flower (GF).

‘XiangLei’, a local main cultivar of *L. macranthoides* in Hunan province, China, identified by Chen et al. [41], was used as material. The fresh flower buds or opening flowers were sampled respectively at the four developmental stages: green flower bud (GB, green, rod-shaped, 2–3 cm long), white flower bud (WB, white, rod-shaped, 4–5 cm long), white flower (WF, white, fully open flower) and golden flower (GF, golden yellow, fully open flower ) (Fig. 9). The samples were immediately frozen in liquid nitrogen and stored at -80℃ until further use in metabolite, RNA sequencing and qPCR analysis. Three biological replicates were performed for each sample.

### Complementary DNA library construction and RNA sequencing

Total RNA was extracted from flower buds or flowers using a plant RNA extraction kit (HUAYUE YANG, Inc. Beijing, China). The quantity and quality of the RNA samples were verified using Nucleic acid/protein analyzer (Eppendorf, Germany). Twelve libraries were constructed and sequenced using Illumina HiSeq. 4000 platform by Genepioneer Biotech Corporation (Nanjing, China).

### Quantitative real-time PCR (qPCR) analysis

To compare the expression data between RNA-seq and qPCR, the relative expression level was transformed to log_2_ (fold change). Screening of DEGs and the qPCR analysis were done as that reported by Lv et al. [2]. The *18 S rRNA* was used as an internal control for normalization of gene expression [42]. The gene-specific primers are listed in supplementary Table S1.

### Flavonoids extraction and analysis

The sample preparation, extraction, identification, quantification and analysis of flavonoids were performed at Metware Biotechnology Co., Ltd. (www.metware.cn, Wuhan, China) following its standard procedures and previously fully described by Pan et al. [24] and Xia et al. [43] respectively. The sample solutions were analyzed by an ultra performance liquid chromatography system (UPLC, SHIMADZU Nexera X2) and a tandem mass spectrometry (MS/MS) system (Applied Biosystems 6500 QTRAP) [24]. Variable importance in projection (VIP) ≥ 1 combined with|log_2_ (fold change)|≥1 and the False Discovery Rate ≤ 0.05 was used to screen DAFs.

### Correlation analysis between metabolome and transcriptome

Correlation analysis was performed using quantitative values of transcripts and metabolites in all samples by calculating Pearson’s correlation coefficients (PCC). The coefficients were calculated from log_2_ (fold change) of each transcript and metabolite with the Excel 2023. Correlations with a|PCC|>0.8 and *p* value < 0.05 were selected.

### Statistical analysis

All determinations were conducted in triplicate, and results are presented as mean ± standard deviation.

### Electronic supplementary material

Below is the link to the electronic supplementary material.

Supplementary Material 1

## Data Availability

The datasets supporting the conclusions of this article has been deposited in the China National GeneBank DataBase (CNGBdb, https://db.cngb.org/) with accession code CNP0003782 or is included within the additional files.

## Abbreviations

DAF: Differentially accumulated flavonoid
DEG: Differentially expressed gene
GO: Gene ontology
LOG: Luteolin-7-O-(6’’-malonyl)-glucoside
K3OG: Kaempferol-3-O-(6’’-O-acetyl)-glucoside
K3OGG: Kaempferol-3-O-(6’’-acetyl) glucosyl-(1→3)-galactoside
GB: Green flower bud
WB: White flower bud
WF: White flower
GF: Golden flower
VIP: Variable importance in projection
PCC: Pearson’s correlation coefficients

## References

[1] Zhang J, Xu BH, Pan X (2019). Study on the distinguishing points of Honeysuckle and Lonicerae Flos. Beijing J Tradit Chin Med.

[2] Lv LL, Feng XF, Li W, Li K (2019). High temperature reduces peel color in eggplant (*Solanum melongena*) as revealed by RNA-seq analysis. Genome.

[3] Treutter D. Significance of flavonoids in plant resistance and enhancement of their biosynthesis. Plant Biol. 2005; 7, 581–91. PMID:16388461. 10.1055/s-2005-873009 10.1055/s-2005-873009 16388461

[4] Bai ZJ, Ren QL, Feng TT, Zhao Z, Zhou Y, Lin B (2015). Study on *Lonicera macranthoides* Hand-Mazz anti-inflammatory activity based on inflammatory cell model. Chin Med J Res Prac.

[5] Wang ZH, Zhou XR, Cheng HZ, Wang Z, Tong QZ, Zhou RB* (2022). Liu X.D*. Prediction of antioxidant and antibacterial quality markers of *Lonicerae Flos* based on multivariate statistical analysis. J Hunan Univ Chin Med.

[6] Wang L, Yang R, Yuan B, Liu Y, Liu C (2015). The antiviral and antimicrobial activities of licorice, a widely-used Chinese herb. Acta Pharm Sin B.

[7] Jones DJL, Lamb JH, Verschoyle RD, Howells LM, Butterworth M, Lim CK, Ferry D, Farmer PB, Gescher AJ (2004). Characterisation of metabolites of the putative cancer chemopreventive agent quercetin and their effect on cyclo-oxygenase activity. Br J Cancer.

[8] Qiao ZQ, Wang XM, Zeng HJ, Liu SS, Cai N, Peng JQ, Huang YZ, Li YX (2021). Clone and expression analysis of *Lm4CL* in *Lonicera macranthoides* Hand-Mazz. J Cent South Univ Forestry Technol.

[9] National Pharmacopoeia Committee. Chinese charmacopoeia. 2015 ed.; China Medical Science and Technology Press, Beijing, China, 2015; pp30-31.

[10] Liu WJ, Chen Y, Ma X, Feng X, Liang JY (2013). Progress in the researcn on chemical constituents of *Lonicera macranthoides* Hand.- Mazz. Chin Wild Plant Resour.

[11] Jia XD, Feng X, Zhao XZ, Wang M, Sun H, Dong YF (2008). Study on chemical constituents from *Lonicera macranthoides*. Chin Traditional Herb Drugs.

[12] Mei YD, Li HB, Wang ZZ, Yu Y, Yao XS, Xiao W (2020). Glycosides from flower buds of *Lonicera macranthoides* [J]. Chin Traditional Herb Drugs.

[13] Chen J, Xu XF, Chai XY, Li P (2006). Chemical constituents in the buds of *Lonicera macranthoides*. Chin J Nat Med.

[14] Szankowski I, Flachowsky H, Li H, Halbwirth H, Treutter D, Regos I, Hanke MV, Stich K (2009). Fischer T.C. Shift in polyphenol profile and sublethal phenotype caused by silencing of anthocyanidin synthase in apple (*Malus* Sp). Planta.

[15] Li H. Cloning and functional analysis of the key enzyme genes involved in the flavonoids biosynthesis in *Salvia Miltiorrhiza*. Doctor’s thesis. Northwest A &F University, Yangling, 2019.

[16] Falcone Ferreyra ML, Rius SP, Casati P (2012). * flavonoids: biosynthesis, biological functions, and biotechnological applications. Front Plant Sci.

[17] Chen Z, Tang N, You Y, Lan J, Liu Y, Li Z (2015). Transcriptome analysis reveals the mechanism underlying the production of a high quantity of vhlorogenic acid in young leaves of *Lonicera macranthoides* Hand.-Mazz. PLoS ONE.

[18] Chen Y, Wang A, Liu C, Long Y, Liu X, Zeng J, Li C, Liu X, Zhou R (2020). Cloning and expression patterns of C3H1 gene in and its correlation with chlorogenic acid content. Chin J Exp Tradit Med Formulae.

[19] Yao T, Wu H, He J (2022). Isolation and identification of chemical constituents in *Lonicerae Flos*. Chin Pharm.

[20] Xue Q, Fan H, Yao F, Cao X, Liu M, Sun J. Liu Y*. Transcriptomics and targeted metabolomics profilings for elucidation of pigmentation in *Lonicera japonica* flowers at different developmental stages. Ind Crop Prod. 2020;145:111981. 10.1016/j.indcrop.2019.111981

[21] Chen C, Zhou G, Chen J, Liu X, Lu X, Chen H, Tian Y (2021). Integrated metabolome and transcriptome analysis unveils novel pathway involved in the formation of yellow peel in Cucumber. Int J Mol Sci.

[22] Yuan Y, Zuo J, Zhang H, Li R, Yu M, Liu S (2022). Integration of transcriptome and metabolome provides new insights to flavonoids iosynthesis in *Dendrobium Huoshanense* Front. Plant Sci.

[23] Yi† D, Zhang† H, Lai B, Liu L, Pan X, Ma Z, Wang Y, Xie J, Shi S*, Y* W. Integrative analysis of the coloring mechanism of red Longan pericarp through metabolome and transcriptome analyses. J Agric Food Chem. 2020;69(6). 10.1021/acs.jafc.0c05023. 10.1021/acs.jafc.0c05023 33332135

[24] Pan Y, Zhao X, Wu X, Wang Y, Tan J, Chen D (2021). Transcriptomic andmetabolomic analyses provide insights into the biosynthesis of chlorogenic acids in *Lonicera macranthoides* Hand.-Mazz. PLoS ONE.

[25] Kanehisa M, Goto SKEGG (2000). Kyoto Encyclopedia of genes and genomes. Nucleic Acids Res.

[26] Yang S, Xu Y, Xing Y, Shi B (2019). Research advances on effects of plant-based flavonoids oniImmune and antioxidative functions in animals. Chin J Anim Nutr.

[27] Shukla S, Gupta S, Apigenin (2010). A promising molecule for cancer prevention. Pharm Res.

[28] Chang CY, Lin TY, Lu CW, Wang CC, Wang YC, Chou SS, Wang SJ (2015). Apigenin, a natural flavonoid, inhibits glutamate release in the rat hippocampus. Eur J Pharmacol.

[29] Ren B, Qin W, Wu F, Wang S, Pan C, Wang L, Zeng B, Ma S, Liang J (2016). Apigenin and naringenin regulate glucose and lipid metabolism, and ameliorate vascular dysfunction in type 2 diabetic rats. Eur J Pharmacol.

[30] Kim JD, Liu L, Guo W, Meydani M (2006). Chemical structure of flavonols in relation to modulation of angiogenesis and immune-endothelial cell adhesion. J Nutr Biochem.

[31] Lee BH, Jeong S, Lee J, Kim J, Yoon IS, Choi SH, Lee S, Chang C, Kim H (2005). Han Y.S. Quercetin inhibits the 5-hydroxytryptamine type 3 receptor-mediated ion current by interacting with pre-transmembrane domain I. Mol Cells.

[32] Owens Dk, Alerding AB, Crosby KC, Bandara AB, Westwood JH (2008). Winkel B.S.J. Functional analysis of a predicted flavonol synthase gene family in Arabidopsis. Plant Physiol.

[33] Nguyen NH, Kim JH, Kwon J, Jeong CY, Lee W, Lee D, Hong SW, Lee H (2016). Characterization of *Arabidopsis thaliana FLAVONOL SYNTHASE 1* (*FLS1*)-overexpression plants in response to abiotic stress. Plant Physiol Bioch.

[34] Winkel-shirley B (2001). A colorful model for genetics, biochemistry, cell biology, and biotechnology. Plant Physio.

[35] Yonekura-Sakakibara SK, Nakabayashi K, Higashi R, Yamazaki Y, Tohge M (2013). Fernie A.R. The flavonoid biosynthetic pathway in Arabidopsis: structural and genetic diversity. Plant Physiol Biol.

[36] Wang Z, Cui Y, Vainstein A, Chen S, Ma H (2017). Regulation of fig (Ficus carica L.) fruit color: metabolomic and transcriptomic analyses of the flavonoid biosynthetic pathway. Front Plant Sci.

[37] Wang Y, Dou Y, Wang R, Guan X, Hu Z, Zheng J (2017). Molecular characterization and functional analysis of chalcone synthase from *Syringa oblata* Lindl. In the flavonoid biosynthetic pathway. Gene.

[38] Qin XY, Qiao JJ, Li Y (2019). N*. Structure, function and application of hydroxycinnamoyl transferase. Chin J Biochem Mol Biol.

[39] Baucher M, Halpin C, Petit-Conil M, Boerjan W (2003). Lignin: genetic engineering and impact on pulping. Crit Rev Biochem Mol Biol.

[40] Comino C, Hehn A, Moglia A, Menin B, Bourgaud F, Lanteri S 1, Portis E. The isolation and mapping of a novel hydroxycinnamoyl transferase in the globe artichoke chlorogenic acid pathway. BMC Plant Biol. 2009;9(1):30. 10.1186/1471-2229-9-30 10.1186/1471-2229-9-30 PMC2664813 19292932

[41] Chen Y, Zhou RB, Pan QP, Tong QZ, Liu XD, Ren MQ (2008). Study on HPLC finger-print of ’xianglei’ *Lonicera macranthoides* in Hunan Province. China Mod Doctor.

[42] Cai J, Zhu Y, Xie S, Chen L, Ouyang L, Liu F, Zhang Y, Liu X, Ttong Q, Yi G (2016). Screening of reference genes in *Lonicera macranthoides* and spatio-temporal expression analysis of *LmAGL15* in MADs-box family. Chin Tradit Herb Drugs.

[43] Xia Y, Chen W, Xiang W, Wang D, Xue B, Liu X, Xing L, Wu D, Wang S. Guo Q*., Liang G*. Integrated metabolic profiling and transcriptome analysis of pigment accumulation in *Lonicera japonica* flower petals during color-transition. BMC Plant Biol. 2021;21:98. 10.1186/s12870-021-02877-y 10.1186/s12870-021-02877-y PMC7890969 33596836

