# Correction: Transcriptomic and targeted metabolomic analyses provide insights into the flavonoids biosynthesis in the flowers of *Lonicera macranthoides*

**DOI:** 10.1186/s12896-024-00860-7

**Published:** 2024-05-20

**Authors:** Ling Ling Lv, Li Yun Li, Long Qian Xiao, Jian Hui Pi

**Affiliations:** Key Laboratory of Research and Utilization of Ethnomedicinal Plant Resources of Hunan Province, Key Laboratory of Hunan Higher Education for Western Hunan Medicinal Plant and Ethnobotany, Huaihua University, Huaihua, 418008 China

**Correction** **:** **BMC Biotechnol 24, 19 (2024)**

**https://doi.org/10.1186/s12896-024-00846-5**

Following publication of the original article [1], the authors identified an error in the sample names of the Figure 6.

The incorrect figure 6 is:

**Fig. 6:**
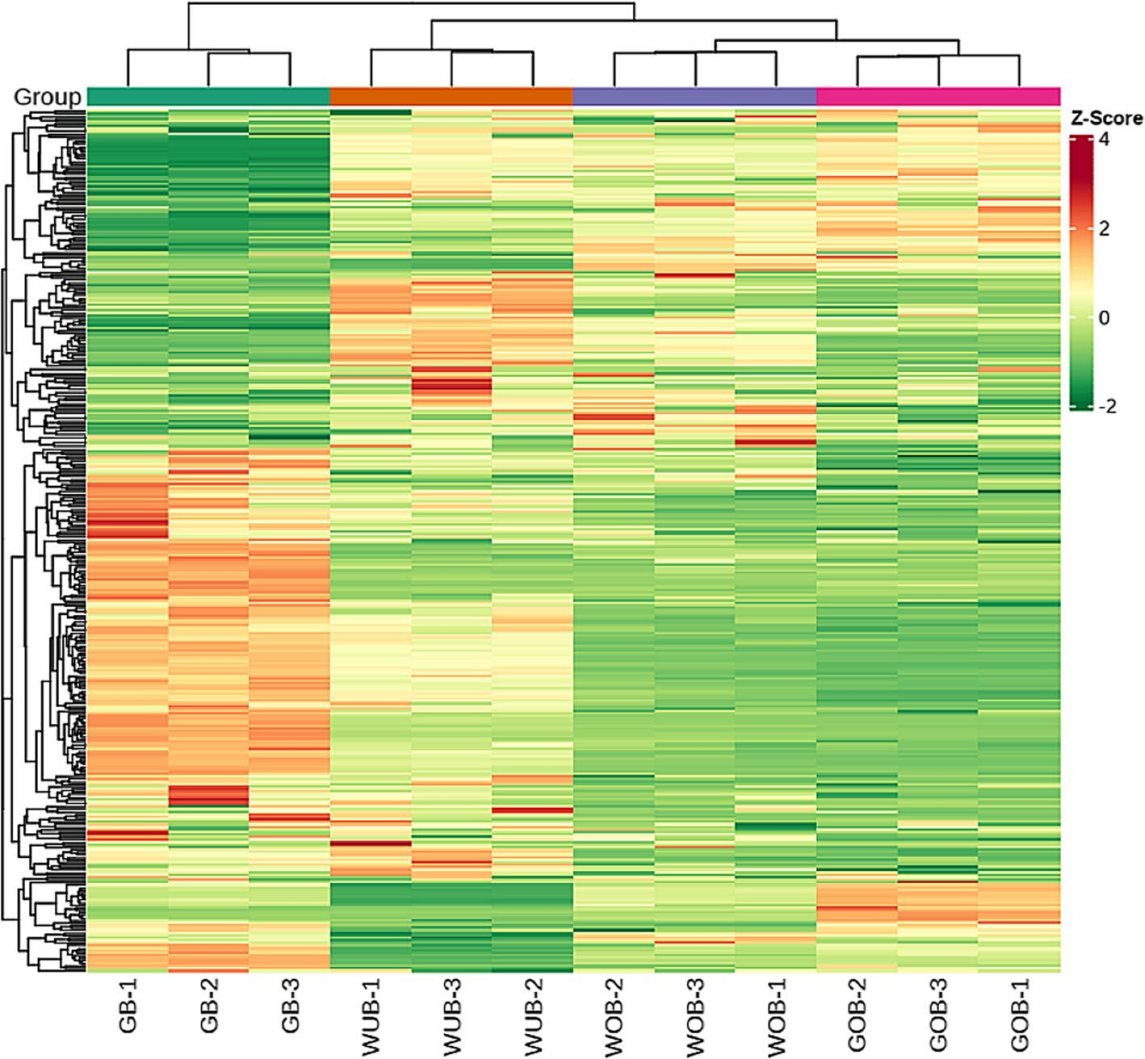
Clustering heat map of the flavonoids detected in the total samples. Each sample is visualized in a single column, and each metabolite is represented in a single row. Red indicates high abundance of metabolites, while green indicates low abundance. GB, green flower bud; WB, white flower bud; WF, white flower; GF, golden flower

The correct figure 6 is:

**Fig. 6:**
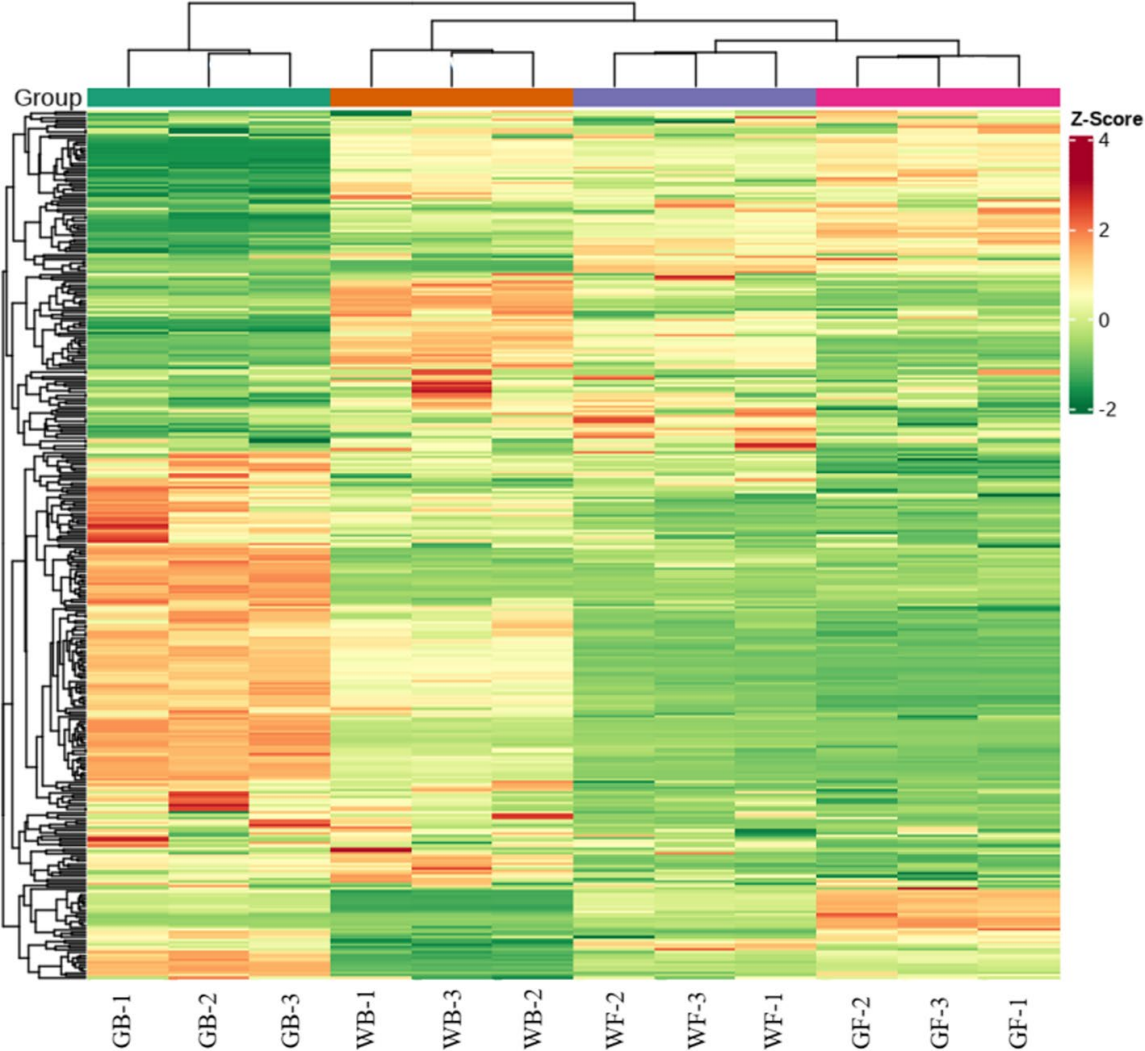
Clustering heat map of the flavonoids detected in the total samples. Each sample is visualized in a single column, and each metabolite is represented in a single row. Red indicates high abundance of metabolites, while green indicates low abundance. GB, green flower bud; WB, white flower bud; WF, white flower; GF, golden flower

The original article has been corrected.
