# Supplementary figures and images for: Transcriptomic and targeted metabolomic analyses provide insights into the flavonoids biosynthesis in the flowers of Lonicera macranthoides
Source: BMC Biotechnol. 2024 Apr 12;24:19. doi: 10.1186/s12896-024-00846-5 (PMC11015657; doi:10.1186/s12896-024-00846-5)

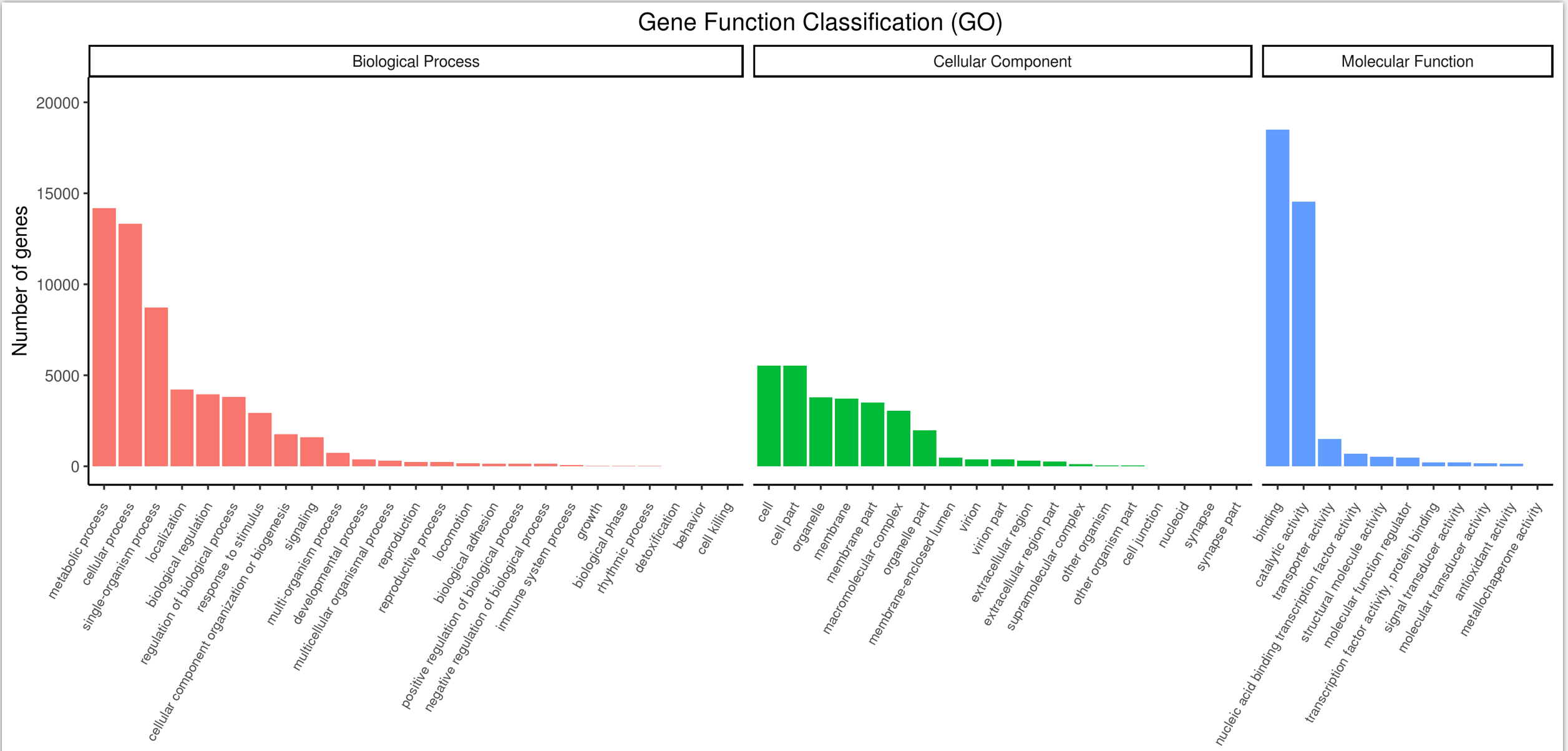


Fig S1 GO classification analysis of unigenes

Supplement: Supplementary file 1 — Supplementary Material 1 [file 12896_2024_846_MOESM1_ESM.docx]
